# iRSpot-DACC: a computational predictor for recombination hot/cold spots identification based on dinucleotide-based auto-cross covariance

**DOI:** 10.1038/srep33483

**Published:** 2016-09-19

**Authors:** Bingquan Liu, Yumeng Liu, Xiaopeng Jin, Xiaolong Wang, Bin Liu

**Affiliations:** 1 School of Computer Science and Technology, Harbin Institute of Technology, Harbin, Heilongjiang 150080, China; 2 School of Computer Science and Technology, Harbin Institute of Technology Shenzhen Graduate School, Shenzhen, Guangdong 518055, China; 3 School of Computer Science and Technology, Harbin Engineering University, Harbin, Heilongjiang 150001, China; 4 Key Laboratory of Network Oriented Intelligent Computation, Harbin Institute of Technology Shenzhen Graduate School, Shenzhen, Guangdong 518055, China

## Abstract

Meiotic recombination presents an uneven distribution across the genome. Genomic regions that exhibit at relatively high frequencies of recombination are called hotspots, whereas those with relatively low frequencies of recombination are called coldspots. Therefore, hotspots and coldspots would provide useful information for the study of the mechanism of recombination. In this study, we proposed a computational predictor called iRSpot-DACC to predict hot/cold spots across the yeast genome. It combined Support Vector Machines (SVMs) and a feature called dinucleotide-based auto-cross covariance (DACC), which is able to incorporate the global sequence-order information and fifteen local DNA properties into the predictor. Combined with Principal Component Analysis (PCA), its performance was further improved. Experimental results on a benchmark dataset showed that iRSpot-DACC can achieve an accuracy of 82.7%, outperforming some highly related methods.

Meiotic recombination is the process alleles exchange between homologous chromosomes during meiosis^1^,^2^. It plays an important role in the process of genome evolution^3^,^4^. Since recombination can produce diverse gametes, so it provides material for natural selection. Moreover, Recombination also influences the genome evolution via gene conversion or mutagenesis^5^,^6^.

Although the mechanism of recombination is still unclear, it has been assured that recombination plays an important part in promoting genome evolution. The distribution pattern of recombination position has drawn much attention and several studies have been performed on chromosomes^7^,^8^,^9^. Some studies have found that recombination presents an uneven distribution across the genome. Genomic regions that exhibit at relatively high frequencies of recombination are called hotspots, while those with relatively low frequencies of recombination are called coldspots^10^,^11^. In the era of rapid development of biology sequencing technology, the number of sequenced genome shows explosive growth. Therefore, it is necessary to develop stable methods for the identification of recombination spots.

Although a great deal of recombination information can be acquired from experiments concerning recombination, identifying recombination hot/cold spots by using the information of DNA sequence is still a challenging task. Recently, several models have been proposed to predict recombination hotspots and coldspots. For example, Zhou *et al*.^12^ proposed a SVM-based method based on codon composition to identify hotspots from coldspots. Later, Jiang *et al*.^13^ employed the Random Forest classifier trained with the gapped dinucleotide composition features to identify hotspots from coldspots in *Saccharomyces cerevisiae*. Guo *et al*.^14^ proposed a SVM model based on DNA physical properties to predict hot/cold spots in yeast. Combining increment of diversity with quadratic discriminant analysis (IDQD), Liu *et al*.^1^ presented a model based on sequence k-mer frequencies along with DNA sequences. Wu *et al*.^15^ proposed a SVM model based on the features of genomic and epigenomic to predict meiotic recombination hotspots in human and mouse. Chen *et al*.^16^ presented a SVM model based on pseudo dinucleotide composition. Wang *et al*.^17^ proposed a method based on gapped kmers. Most of these predictors only considered the local sequence-order information, while little global sequence-order information was taken into account. However, in many bioinformatics’ tasks, the global sequence-order information has showed strong discriminative power as shown in many studies. Therefore, in a predictor, the global sequence-order factor should be incorporated. Unfortunately, it is not an easy job, because the lengths of DNA sequences are different.

To address this problem, a feature called dinucleotide-based auto-cross covariance (DACC)^18^ is applied to recombination hot/cold spots identification, which is able to incorporate the global sequence-order effects in the DNA sequences into the predictor. Combined with Support Vector Machines (SVMs), a predictor called iRSpot-DACC is proposed. Later, in order to further improve its performance and computational cost, Principal Component Analysis (PCA)^19^ is adopted. Experimental results on a benchmark dataset demonstrate that the proposed method outperformed some highly related models, including IDQD^1^ and iRSpot-PseDNC^16^.

## Results

### Influence of parameters on the predictive performance of iRSpot-DACC

**Figure 1:**
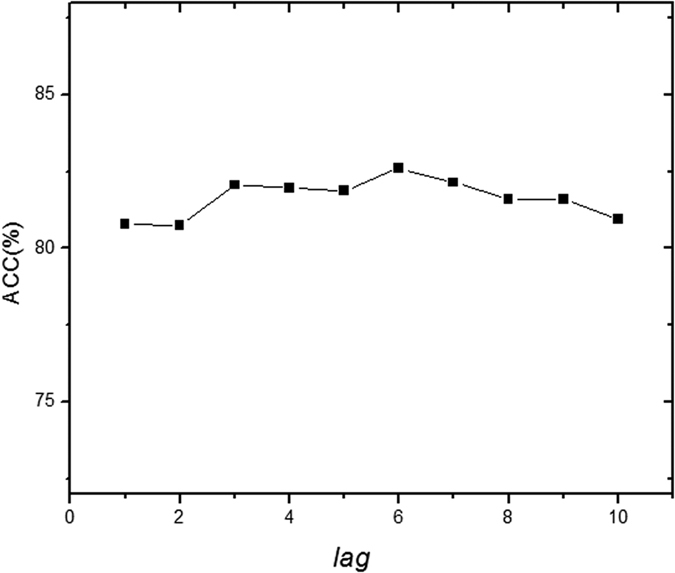
The distribution of Acc values achieved by iRSpot-DACC with different *lag* values based on the benchmark dataset through five-fold cross validation.

In iRSpot-DACC, there is a parameter, the distance between two dinucleotides *lag*, would affect its predictive performance. In the current study, *lag* is optimized via the 5-fold cross validation. The influence of *lag* on the performance of iRSpot-DACC is shown in Fig. 1, from which we can see that the optimized value can be achieved when *lag* = 6, and this parameter has little impact on the performance. DACC is the combination of Dinucleotide-based auto covariance (DAC) and Dinucleotide-based cross covariance (DCC) (*cf.* section Material and Methods). With this parameter setting, the lengths of the feature vectors for DAC and DCC are 15 × 6 = 90 and 15 × 14 × 6 = 1260 respectively. Therefore, the dimension of DACC is 90 + 1260 = 1350.

### The computational performance of iRSpot-DACC can be further improved by using PCA

In order to further improve its performance and computational cost of iRSpot-DACC, the Principal Component Analysis (PCA)^19^ is employed.

There is a parameter *w (cf.* Eq. (18)) in PCA, which would have impact on both the predictive accuracy and the dimension of the feature vectors. Therefore, we optimize this parameter utilizing 5-fold cross validation. The results show that the iRSpot-DACC-PCA (iRSpot-DACC combined with PCA) achieves the best performance when *w* = 0.99 and its performance is shown in Table 1, from which we can see that iRSpot-DACC-PCA outperforms iRSpot-DACC.

The feature vector’s dimension of iRSpot-DACC-PCA is 173, which is significantly smaller than the original dimension of iRSpot-DACC (1350). Therefore, the predictive accuracy and the computational cost of iRSpot-DACC are further improved by using PCA.

### Discriminative visualization and interpretation

In order to further explore the discriminative power and indicate the meaning of the feature space in biology, we calculate the discriminative weight vector according to the study^20^. The specific formula of the feature discriminative weight vector **W** can be formulated as:


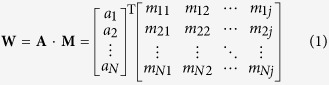


where **A** is the specific weight for each training samples obtained from SVM training process; **M** is the feature space of the benchmark dataset used in the current study; **N** is the number of DNA sequences in the training dataset; *j* is the dimension of the feature vector. Therefore, **W** is a 1 × *j* vector and each element in it represents the corresponding feature’s discriminative power.

**Figure 2:**
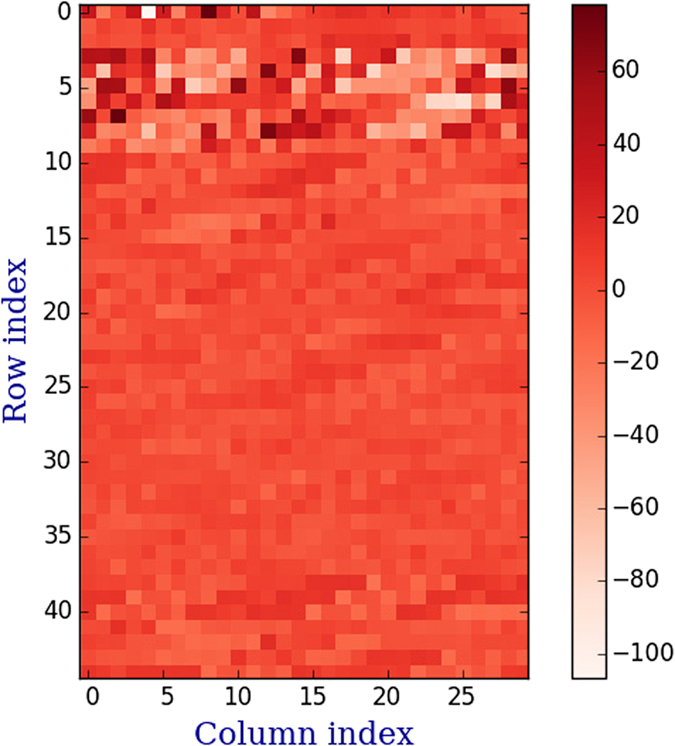
An illustration for the discriminant visualization. The figure labeled by *y*-axis and *x*-axis shows the distribution of different features. The *adjacent color bar* shows the mapping of sum score values.

The feature discriminative weight vector with 1350 features (*cf.* section Results) is depicted in Fig. 2, in which the deeper color spots represent stronger discriminative power than the lighter color spots. From Fig. 2 we can see that the top three discriminative features are DAC(2, 3), DCC(2, 8, 3) and DCC(2, 15, 1). All the three features are deduced from the same property (F-tilt), which suggests the importance of this property of F-tilt (*μ* = 2). The top ten discriminative features are listed in Table 2. In this table, we can conclude several conclusions. First, the correlation between properties F-roll (*μ* = 1) and several other properties shows strongly discriminative power for identifying recombination hot/cold spots. Second, the correlation between F-tilt (*μ* = 2) and other properties including itself also shows strongly discriminative power. Third, when the distance between two dinucleotides equals to 1, 2, 3 or 5, the influence of the corresponding features would be important for identifying hot/cold spots.

### Comparison with other related predictors

Two methods for hot/cold spots identification are compared with the proposed methods iRSpot-DACC and iRSpot-DACC-PCA, including IDQD^1^ and iRSpot-PseDNC^16^. The results of various methods on the benchmark dataset **S** are shown in Table 1.

According to Table 1, we can see that iRSpot-DACC outperforms the two methods IDQD^1^ and iRSpot-PseDNC^16^. Furthermore, iRSpot-DACC-PCA outperforms iRSpot-DACC by adopting Principal Component Analysis (PCA). The main reasons are described as follows: IDQD^1^ only consider the local sequence-order information, and iRSpot-PseDNC^16^ improves it by incorporating global sequence-order information. However, iRSpot-DACC not only incorporates the global sequence-order information but also contains more DNA properties into the feature vectors. Therefore, we conclude that iRSpot-DACC would be a useful tool for hot/cold spots identification.

## Discussion

In this study, we propose a computation method called iRSpot-DACC for yeast hot/cold spots identification. The method incorporates long range or global sequence-order information. The result shows that iRSpot-DACC outperform other state-of-the-art predictors. Furthermore, iRSpot-DACC incorporates the correlations between different dinucleotide DNA properties. Another important advantage of our approach derived from PCA (principal component analysis)^21^ which not only can improve the predictive accuracy, but also can reduce the computational cost. It can be expected that DACC would be a powerful feature extraction method, and it can be applied to other tasks in the field of bioinformatics, such as DNA-binding proteins identification^22^, protein fold prediction^23^,^24^, cytokine detection^25^,^26^, protein-protein interaction site prediction^27^, tumor classification and analysis^28^, etc. Moreover, since publicly accessible web-server is beneficial to develop more useful predictors, we would make efforts in our future work to develop a web-server for the method proposed in this paper. Furthermore, we will apply other advanced machine learning techniques to establish more accurate predictors for hot spot identification, such as deep learning, and neural networks^29^,^30^,^31^,^32^.

## Material and Methods

### Benchmark Dataset

The benchmark dataset used in this study was constructed by Jiang *et al*.^13^, which contains 490 hotspots and 591 coldspots. For more detailed information of this benchmark dataset, please refer to^13^.

Therefore, the benchmark dataset for the current study can be expressed as:


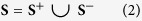


where **S**^+^ is the set of recombination hotspots, **S**^−^ is the set of recombination coldspots, and 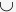 is a mathematical operator representing “union”.

### Dinucleotide-based auto-cross covariance (DACC)

As described above, the global sequence-order information shows strongly discriminative power for identifying recombination hot/cold spots. Therefore, it is crucial to incorporate the global sequence-order information into our model. In order to deal with this problem, a feature called Dinucleotide-based auto-cross covariance (DACC)^18^ is adopted, which incorporates global sequence-order information along DNA sequences. DACC is the combination of Dinucleotide-based auto covariance (DAC) and Dinucleotide-based cross covariance (DCC). Next, we will introduce DAC and DCC respectively.

Given a DNA sequence **D**


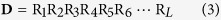


where *L* is the length of DNA sequence, R_1_ means the nucleic acid residue at the first position in the sequence, R_2_ means the nucleic acid residue at the second position and so forth.

The DAC^18^,^33^,^34^ represents the correlation of one DNA local property between two dinucleotides at a distance of *lag* in the sequence. DAC can be calculated by:


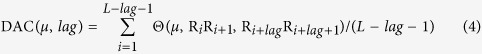


and


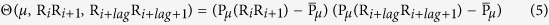


where *μ* is the index of dinucleotide local property; *L* represents the DNA sequence length; P_*μ*_(R_*i*_R_*i*+1_) means the value of the dinucleotide R_*i*_R_*i*+1_ at position *i* for the local property index *μ*; 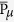 is the average value of P_*μ*_(R_*i*_R_*i*+1_) for a DNA sequence and can be calculated as:


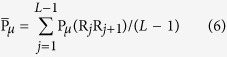


In such way, the feature vector’s length of DAC is N*LAG, where *N* is the number of dinucleotide properties used in this study and *LAG* is the maximum of *lag* 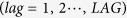.

**Figure 3:**
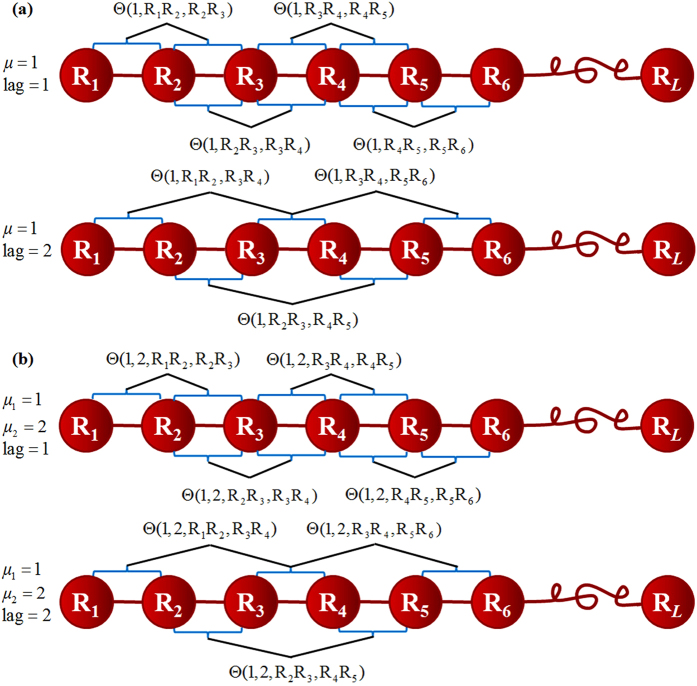
The process of generating DACC feature vector. (**a**) The generating process of DAC feature vector. It depicts the correlation of the same property index between two dinucleotides. (**b**) The generating process of DCC feature vector. It depicts the correlation of the different property indices between two dinucleotides.

The DCC^33^,^34^,^35^ calculates the correlation of two different properties between two dinucleotides at a distance *lag* nucleic acid residues in the DNA sequence. DCC can be calculated by using the following equation:


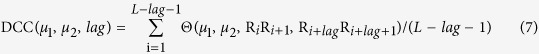


and


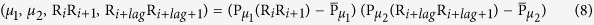


where *μ*_1_, *μ*_2_ are two different property indices, *L* represents the DNA sequence length; P*μ*_1_(R_*i*_R_*i*+1_) P*μ*_2_(R_*i*_R_*i*+1_)) is the numerical value of the dinucleotide (R_*i*_R_*i*+1_) at position *i* for the property index *μ*_1_ (*μ*_2_); 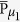 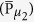 is the average value for property index value *μ*_1_ (*μ*_2_) along the whole sequence and have the same form with Eq. (6). In such way, the feature vector’s length of DCC is *N* * (*N* − 1) * *LAG*, where *N* is the number of dinucleotide properties used in this study and *LAG* is the maximum of *lag* 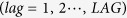. The processes for generating the feature vectors of DAC and DCC are presented in the Fig. 3(a,b) respectively.

In this study, fifteen properties from^36^ are used. Their values are listed in Table 3.

### Support vector machine (SVM)

Support Vector Machine (SVM) is a pattern recognition technique introduced by Vapnik^37^, which has been employed for many computational tasks in bioinformatics^38^,^39^,^40^,^41^. It seeks an optimal hyperplane via transforming the original feature space into a high dimensional vector space to achieve classification.

In the current study, the ANACONDA package (http://www.continuum.io/) is adopted, which contains the implementation of SVM. The selected kernel function is radial basis function (RBF), which is defined as:


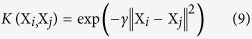


Two parameters, the regularization parameter *C* and the kernel width parameter *γ* are optimized on the dataset by using a grid tool provided by ANACONDA. In the current study, the values of the two parameters are shown below:


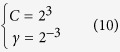


### Principal Component Analysis (PCA)

Feature selections are able to remove the noise so as to improve the classification performance^42^. In order to reduce redundant information, in this study, we adopt Principal Component Analysis (PCA)^19^ to reduce the dimension of the original feature vectors. It reduces the dimension of the feature vectors through projecting a feature space onto a smaller subspace that represents the dataset well.

Suppose, the original feature space of iRSpot-DACC can be represented as:


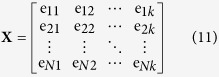


where *N* is the number of training sample, *k* is the dimension of the feature vectors. Then, the averages for every dimension of **X** can be expressed as:


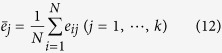


where *N* and *k* have the same meaning with Eq. (11). Therefore, the matrix which is composed of mean vectors for every dimension in **X** can be represented as:


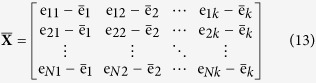


where *e*_*ij*_ represents the element of **X** and 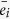 can be acquired from Eq. (12).

Then, the covariance matrix 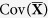 and its eigenvalues can be calculated and the eigenvalues can be represented as:


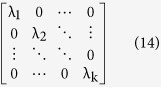


Next*, l* eigenvectors whose corresponding eigenvalues are more bigger than other eigenvectors’ are chosen to form a matrix, which can be represented as:


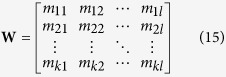


where each column represents an eigenvector and their corresponding eigenvalues can be represented as:


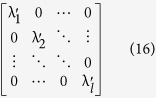


where 
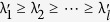
. Finally, the new subspace **M** can be calculated by


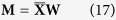


Therefore, the dimension of the feature space is reduced from *k* to *l*. The values of *k* and *l* have been discussed in section Results.

The selection of principal components is based on the cumulative weight ratio *w*:


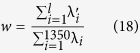


The values of *w* and *l* have been discussed in section Results.

### Jackknife test

In statistical prediction, three cross-validation methods including independent dataset test, sub-sampling (or K-fold cross-validation) test and jackknife test are often used to measure the performance of a predictor^43^,^44^,^45^. Among the three methods, jackknife test is deemed the most objective which urging it to be widely adopted by researchers to evaluate the performance of various classifiers. Therefore, in the current study, jackknife test is also adopted to measure the performance of iRSpot-DACC and iRSpot-DACC-PCA. In the jackknife test, each sequence in the benchmark dataset would be selected as test sample and the corresponding remaining samples as training samples.

### Criteria for performance evaluation

Sensitivity (Se), Specificity (Sp), Accuracy (Acc), and Matthew’s Correlation Coefficient (Mcc)^46^ are used to evaluate the performance of different methods. They are defined as follows:


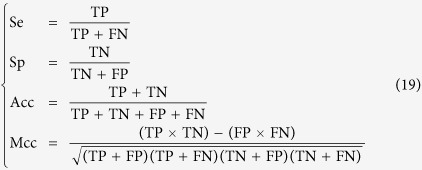


where TP, FP, TN and FN represent the true positive, false positive, true negative and false negative respectively.

## Additional Information

**How to cite this article**: Liu, B. *et al*. iRSpot-DACC: a computational predictor for recombination hot/cold spots identification based on dinucleotide-based auto-cross covariance. *Sci. Rep.* **6**, 33483; doi: 10.1038/srep33483 (2016).

## Figures and Tables

**Table 1:** Results of different predictors on benchmark dataset.

Predictor	Test method	Sn(%)	Sp(%)	Acc (%)	MCC
IDQD^a^	5-fold	79.40	81.00	80.30	0.603
iRSpot-PseDNC^b^	Jackknife	73.06	89.49	82.04	0.638
iRSpot-DACC^c^	Jackknife	75.71	88.16	82.52	0.647
iRSpot-DACC-PCA^d^	Jackknife	76.33	87.99	82.70	0.651

^a^From Liu *et al*.^1^;

^b^From Chen *et al*.^16^;

^c^The parameter used: *lag* = 6 for Eq. (4) and Eq. (7); *C* = 2^3^ and *γ* = 2^−3^ for the LIBSVM^47^;

^d^The parameter used: *lag* = 6 for Eq. (4) and Eq. (7); *C* = 2^3^ and *γ* = 2^−3^ for the LIBSVM^47^; *w* = 0.99 for PCA.

**Table 2:** The top ten most important features in iRSpot-DACC for identifying hot/cold spots.

Features	Parameters			
	*μ*_1_	*μ*_2_	*lag*	Discriminative power
DAC	F-tilt	F-tilt	3	78.56
DCC	F-tilt	tilt	3	77.98
DCC	F-tilt	entropy	1	70.56
DCC	F-roll	F-slide	3	67.56
DCC	F-roll	twist	1	66.33
DCC	F-tilt	F-roll	5	63.03
DCC	F-roll	energy	5	60.57
DCC	F-roll	F-rise	5	59.84
DCC	F-tilt	tilt	1	58.81
DCC	F-roll	rise	2	54.74

*μ*_1_ and *μ*_2_ are the indices of dinucleotide local property, *lag* is the distance between two dinucleotides and the value of discriminative power represents the discriminative power of the corresponding features. The larger the value is, the stronger the discriminative power. The calculation of this value refers to Eq. (1).

**Table 3:** The values of the fifteen DNA dinucleotide properties.

	AA/TT	AC/GT	AG/CT	AT	CA/TG	CC/GG	CG	GA/TC	GC	TA
F-roll	0.04	0.06	0.04	0.05	0.04	0.04	0.04	0.05	0.05	0.03
F-tilt	0.08	0.07	0.06	0.10	0.06	0.06	0.06	0.07	0.07	0.07
F-twist	0.07	0.06	0.05	0.07	0.05	0.06	0.05	0.06	0.06	0.05
F-slide	6.69	6.80	3.47	9.61	2.00	2.99	2.71	4.27	4.21	1.85
F-shift	6.24	2.91	2.80	4.66	2.88	2.67	3.02	3.58	2.66	4.11
F-rise	21.34	21.98	17.48	24.79	14.51	14.25	14.66	18.41	17.31	14.24
roll	1.05	2.01	3.60	0.61	5.60	4.68	6.02	2.44	1.70	3.50
tilt	−1.26	0.33	−1.66	0.00	0.14	−0.77	0.00	1.44	0.00	0.00
twist	35.02	31.53	32.29	30.72	35.43	33.54	33.67	35.67	34.07	36.94
slide	−0.18	−0.59	−0.22	−0.68	0.48	−0.17	0.44	−0.05	−0.19	0.04
shift	0.01	−0.02	−0.02	0.00	0.01	0.03	0.00	−0.01	0.00	0.00
rise	3.25	3.24	3.32	3.21	3.37	3.36	3.29	3.30	3.27	3.39
energy	−1.00	−1.44	−1.28	−0.88	−1.45	−1.84	−2.17	−1.30	−2.24	−0.58
enthalpy	−7.60	−8.40	−7.80	−7.20	−8.50	−8.00	−10.60	−8.20	−9.80	−7.20
entropy	−21.30	−22.40	−21.00	−20.40	−22.70	−19.90	−27.20	−22.20	−24.40	−21.30
